# Analysis of the genome-wide variations among multiple strains of the plant pathogenic bacterium *Xylella fastidiosa*

**DOI:** 10.1186/1471-2164-7-225

**Published:** 2006-09-01

**Authors:** Harshavardhan Doddapaneni, Jiqiang Yao, Hong Lin, M Andrew Walker, Edwin L Civerolo

**Affiliations:** 1 University of California Davis, Department of Viticulture and Enology, Davis, CA 95616, USA; 2 Citrus Research Board, 323 W. Oak, P.O. Box 230, Visalia, CA 93279, USA; 3 USDA-ARS. San Joaquin Valley Agricultural Science Center, 9611 So. Riverbend Ave. Parlier, CA 93648, USA

## Abstract

**Background:**

The Gram-negative, xylem-limited phytopathogenic bacterium *Xylella fastidiosa *is responsible for causing economically important diseases in grapevine, citrus and many other plant species. Despite its economic impact, relatively little is known about the genomic variations among strains isolated from different hosts and their influence on the population genetics of this pathogen. With the availability of genome sequence information for four strains, it is now possible to perform genome-wide analyses to identify and categorize such DNA variations and to understand their influence on strain functional divergence.

**Results:**

There are 1,579 genes and 194 non-coding homologous sequences present in the genomes of all four strains, representing a 76. 2% conservation of the sequenced genome. About 60% of the *X. fastidiosa *unique sequences exist as tandem gene clusters of 6 or more genes. Multiple alignments identified 12,754 SNPs and 14,449 INDELs in the 1528 common genes and 20,779 SNPs and 10,075 INDELs in the 194 non-coding sequences. The average SNP frequency was 1.08 × 10^-2 ^per base pair of DNA and the average INDEL frequency was 2.06 × 10^-2 ^per base pair of DNA. On an average, 60.33% of the SNPs were synonymous type while 39.67% were non-synonymous type. The mutation frequency, primarily in the form of external INDELs was the main type of sequence variation. The relative similarity between the strains was discussed according to the INDEL and SNP differences. The number of genes unique to each strain were 60 (9a5c), 54 (Dixon), 83 (Ann1) and 9 (Temecula-1). A sub-set of the strain specific genes showed significant differences in terms of their codon usage and GC composition from the native genes suggesting their xenologous origin. Tandem repeat analysis of the genomic sequences of the four strains identified associations of repeat sequences with hypothetical and phage related functions.

**Conclusion:**

INDELs and strain specific genes have been identified as the main source of variations among strains, with individual strains showing different rates of genome evolution. Based on these genome comparisons, it appears that the Pierce's disease strain Temecula-1 genome represents the ancestral genome of the *X. fastidiosa*. Results of this analysis are publicly available in the form of a web database.

## Background

*Xylella fastidiosa *(Xf) is responsible for causing economically important diseases in grapevine, citrus and many other plant species. Foremost among these are Pierces's disease (PD) of grapevine, citrus variegated chlorosis (CVC), and leaf scorch diseases in almond (ALS) and oleander (OLS) [1-3]. Due to its potential threat to US agriculture, *X. fastidiosa *(CVC strain) is included in the Federal government's select agent list [4]. The CVC strain (9a5c) was the first plant pathogenic bacterium whose genome was completely sequenced [5]. This was followed by publication of draft sequences of the genomes of Dixon (almond) and Ann1 (oleander) [6] and the complete sequence of the genome of the PD-associated Temecula-1 strain [7]. To date, emphasis of the above published research has been on the functional reconstruction and deciphering the metabolic pathways.

Comparative genome sequencing of bacteria is a powerful means of detecting sequence diversity among closely related, but distinct populations. Comparative whole-genome information about strain specific DNA variation will have important implications for the development of new molecular markers for detection, pathovar classification, disease epidemiology and understanding evolutionary relationships. Using whole genome sequences of four *X. fastidiosa *strains, we conducted sequence analyses for genome-wide DNA- based variations that presumably are critically important in strain divergence, host specificity and pathogenicity.

Currently, genetic variation using markers such as the 16S-23S rRNA spacer region [8], simple sequence repeat markers or Variable Number of Tandem Repeats (VNTRs) that are capable of differentiating among, and within, host-associated strains exists [9,10]. However, information on DNA based variations in the coding and non-coding regions and information on SNPs (Single Nucleotide Polymorphisms) and insertions/deletions (INDELs) of one to several hundred base pairs, thus far have not been studied. Such information is extremely valuable for understanding the epidemiology of this bacterium which has specific host preference and pathogenicity [11]. In nature, pathogen populations with high genetic diversity have high evolutionary potential and thus are more likely to overcome host genetic resistance than pathogen populations with low genetic diversity. The resulting changes in population structure or virulence can lead to resistance breakdown. This is particularly true in agricultural production systems in which mono-culture is a common practice. Under these conditions, the frequency of pathogen genotypes with increased virulence may increase and ultimately lead to resistance breakdown and increased disease incidence. Therefore, availability of such genomic information on coding and non-coding- polymorphic loci will help in linking variability in pathogenicity of different strains to differences in their genetic backgrounds and monitoring changes in their genetic diversity.

INDELs are important events in establishing genomic variations between similar strains [12]. There are numerous mechanisms by which INDELs are formed, such as the DNA recombination, expansion of repetitive DNA sequences and insertion sequence (IS)-mediated events. INDELs serve as reliable signature sequences and have a definite advantage over the traditional phylogenetic analyses based on the gene or protein sequences due to the fact that the traditional analysis derives phylogenetic relationships assuming constant mutation frequency, which is incorrect over long periods of time, leading to incorrect species relationships [13]. On the other hand, conserved INDELs of defined sizes are not greatly affected by such differences in evolutionary rates [14]. Among bacterial species, INDELs have been identified as the principal source of genome variability in *Mycobacterium tuberculosis *complex [15].

Another important factor that contributes to genomic variations is the occurrence of Single Nucleotide polymorphs (SNPs). SNPs have extremely low mutation frequency and are less prone to homoplasty when compared to other molecular markers, making them extremely valuable for phylogenetic analyses. SNPs have been effectively used in drawing evolutionary relationships of *Bacillus anthracis*, the causative organism of anthrax, with extremely low strain variability [16]. A total of 990 SNP markers genome-wide were used in their study. Recently, SNPs were found to be of invaluable source in tracing the worldwide spread of pathogenic *Mycobacterium leprae*, the causative organism of leprosy [17]. Apart from the phylogenetic analysis, SNPs have been identified as functional tools in linking the DNA variations in the promoter of the nitrate reductase gene cluster narGHJI to the observed differences in the nitrate reductase activity of *M. tuberculosis *and *M. bovis *[18] and in showing a link between DNA variability in the *gyrA *gene to *Salmonella enterica *strains resistance to quinolones [19].

There are several means by which bacteria can acquire genes: conjugal transfer, phage-mediated insertions and the uptake of native DNA from the outside sources [20,21]. While not all the genes that are introduced are retained, there are numerous instances where the stable introductions have been shown to play a pivotal role in the evolution of niche-adaptive and pathogenic characteristics of bacterial species, and thus greatly influence inter-strain differences in gene complement [20,22,23]. In certain instances, 10–20% of the genes are estimated to have been laterally transferred [24]. Xenologues have been identified in the past based on criteria, such as G+C content variation (the standard method), codon usage bias and differences in amino acid usage [25].

The present study was undertaken to identify and characterize the macro (present or absent), medium (Tandem repeat variations) and micro (SNPs and INDELs) -level differences from coding and non-coding regions among the four published *X. fastidiosa *strain genomes leading to disease development, and for use in development of improved pathogen diagnostic and epidemiological tools. The results of this study are available through our database.

## Results and discussion

### Global comparative analysis of the *X. fastidiosa *genomes

**Figure 1:**
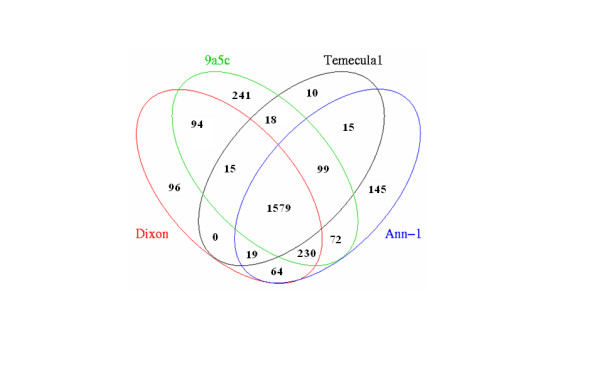
**Summary results of the four-way analysis of the annotated genes among four *Xylella fastidiosa *strains**. There are 1,579 genes that are conserved in all the four strains; the remaining genes show different degrees of overlap. The full sets of results are available at the website [41].

There are 1,579 homologous genes representing 87 families and 194 non-coding sequences in all the four *X. fastidiosa *strains, which account for 76.2% and 3.6%, respectively, of the total genome size. Of these, 108 conserved genes (6.8%) are unique to *X. fastidiosa *(see Additional file 1). The number of strain specific unique genes in each strain varied from 10 to 241 and that of non-coding (intergenic) sequences from 68 to 147 (Fig. 1). The CVC-associated strain 9a5c genome (241 genes) had the highest number of strain specific genes followed by Ann1 (145 genes), Dixon (96 genes) and Temecula-1 (10 genes) genomes (Table 1) (Fig. 1). A fraction of these strain specific genes when BLAST searched against the NCBI database did not show a hit, suggesting that these are also unique to that strain with no known homology to the sequenced bacterial genomes [Temecula-1 (9), Dixon (54), Ann1 (83) and 9a5c (60)].

### Gene families and functional classification

In order to provide a comparable and uniform functional gene classification of all the four strains, the genes were classified based on MIPS [26]. The published 9a5c and Temecula-1 functional assignments [27] were based on a BLAST similarity search followed by the functional assignment similar to the *Escherichia coli *classification [5,7]. On the other hand, the Integrative Genomics [28] classified the other three strains (9a5c, Ann1 and Dixon) using their ERGO suit [6]. This prompted us to use the MIPS functional assignment for all four strains.

**Figure 2:**
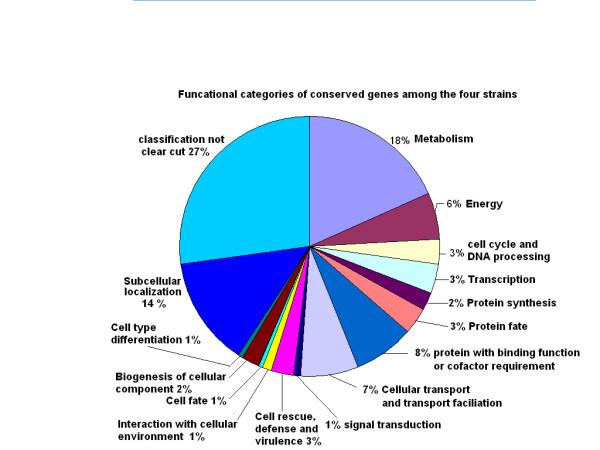
**Functional categories of the 1579 conserved genes among four strains of *X. fastidiosa***. Genes were catalogued according to the MIPS system.

The 1,579 conserved genes could be grouped into 87 functional categories. Further, these 87 categories could be merged to represent 16 major functional categories (Fig. 2). A total of 1,476 (93.5%) of these conserved homologous genes are also present in other organisms. These genes are involved in cellular activities such as metabolism, protein synthesis, cell cycle and other house keeping activities.

### Pair wise SNP and INDEL analysis

There are 12,754 SNPs and 14,449 INDELs in the common genes and 20,779 SNPs and 10,075 INDELs in the 194 non-coding sequences. The average mutation frequency for conserved genes among the six compared groups was 3.93%, which equals to one mutation for every 25 to 26 nucleotides. Pair-wise analysis showed that for strains of 9a5c, Ann1 and Dixon, the lowest mutation frequency was obtained when each of them was compared with Temecula-1, which indicates a closer phylogeny relationship of these strains to Temecula-1. INDELs occurred at 53.1% (812) of the total genes, and SNPs occurred in every gene (Table 2).

The average SNP frequency was 1.08 × 10^-2 ^per base pair, which translates to approximately one SNP for every 93 bp of the DNA. The average INDEL frequency was 2.06 × 10^-2 ^per base pair, which translates to approximately one INDEL for every 30 bp of DNA (Table 2).

**Figure 3:**
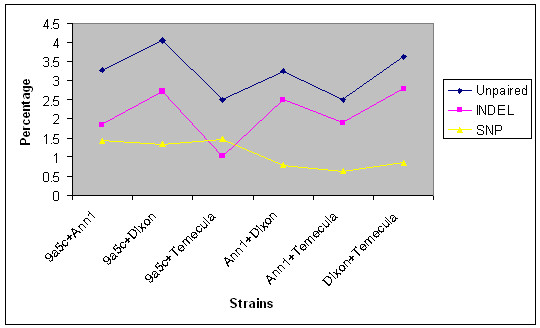
Pair-wise comparison of the 1,528 conserved genes among four sequenced strains of *X. fastidiosa *(9a5c (001), Ann1 (002), Dixon (003) and Temecula-1 (004)) for unpaired nucleotides, INDELs and SNPs.

To identify the relationship of SNPs and INDELs with the overall mutation frequency, a comparison between SNP frequency, INDEL frequency and the overall mutation frequency was carried out (Fig. 3). Clearly, the overall mutation frequency is closely associated with INDEL frequency. On average, 66.50% mutations were due to INDELs with varying degrees among the four strains. The minimum INDEL percentage (1.034%) was found in the genome alignment of 9a5c and Temecula-1, while the maximum (4.049%) was observed in the genome alignment of 9a5c and Dixon (Table 2). Compared to INDELs, the percentage of SNPs was constant for the groups studied. The maximum difference observed was less than 0.7% (Table 2).

**Figure 4:**
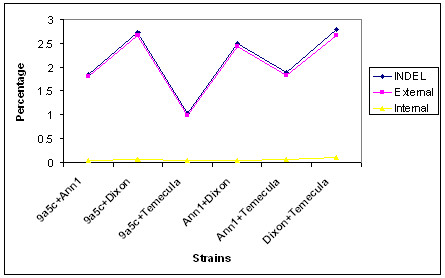
Percentage of external and internal INDELs among the 1,528 conserved genes of four sequenced strains of *X. fastidiosa *(9a5c, Ann1, Dixon and Temecula-1.

Based to their location, two types of INDELs were defined: internal INDELs, located between the start and stop codons; and external INDELs, located before the stop codon and/or after the stop codon. It is interesting that the majority of INDELs (96.7 %) are of external type (Fig. 4). Another interesting finding was that 11.8% (180) of the conserved genes contain internal INDELs whose number is not a multiple of 3, indicating that these INDELs cause frame shift mutations (see Additional file 2).

**Figure 5:**
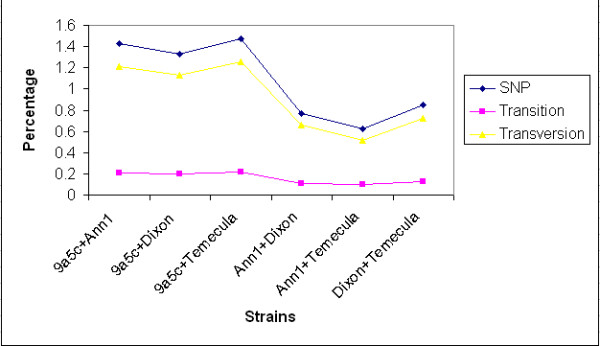
Percentage of SNPs, transitions and transversions among 1,528 conserved genes of four sequenced strains of *X. fastidiosa *(9a5c, Ann1, Dixon and Temecula-1).

SNPs were defined according to their nucleotide types as transversion or transition types. On average, 85.2% of SNPs are transversion type suggesting that transversion was the major type of SNP in *X. fastidiosa *(Fig. 5). Synonymous vs non-synonymous SNPs, were next identified between the above homologous genes. However, for this analysis, those gene pairs that shows internal INDELs that cause frame shift mutations were not included. The results show that on an average, 60.33% of the SNPs cause synonymous changes while 39.67% cause non-synonymous changes (Table 3).

Recently, Lin et al. [10] reported multi-locus *X. fastidiosa *genetic analysis system based on 34 Simple Sequence Repeat (SSR) loci. Diversity analysis based on 83 Xf strains from four geographical locations of the California: Napa, Sonoma, Kern and Riverside counties suggests that genetic differentiation of Xf was partly driven by the host selection. Strain divergence in *X. fastidiosa *was also recently reported using Multilocus Sequence Typing (MLST) of seven to ten loci [29].

Significant and important information regarding strain diversity and dispersal has been obtained using the above markers. However, due to their very nature, the percentage of genome analyzed for drawing the conclusions in these studies is limited. For instance, MLST is currently the most reliable and widely used molecular marker for defining sequence types (ST) among bacterial species. In principle, STs are defined based on the variations in a selected set of housekeeping genes. However, a major limitation factor of MLST stems from the fact that two isolates with the same ST may differ in regions that are subjected to high-frequency transposition or site-specific recombination events that are out side the target locus.

This genome-wide SNP survey is potentially useful as a routine, high-throughput screening tool to identify potential disease outbreaks and appearance of strain variants. Moreover, SNPs offer development of linkage disequilibrium (LD) blocks that are several fold denser that those defined by the Short Tandem Repeats (STRs). Gene-based locus-specific SNPs could provide possible linkage between phenotype and genotype; therefore, such SNPs can be used for functional genotyping.

INDELs play a major role in causing sequence divergence between closely related DNA sequences in animal, plants, insects and bacteria [12]. Our analysis supports this hypothesis. Further, our analysis showed that external INDELs are the major cause for the observed strain differences in *X. fastidiosa*, similar to that observed in the mycobacterial species complex diversification [15]. The observed results suggest that *X. fastidiosa *adapts to its environment by using different transcription initiation points. In 61.01% of the observed internal INDELs, the number of missing nucleotides is not a multiple of three. Such internal INDELs, would result in a frame shift mutation, thus providing another mechanism of gene regulation in this bacterium. Similarly, the four sequenced genomes carry strains specific genes, some of which are unique to those strains. A majority of these genes code for hypothetical proteins followed by gene showing homology to the plasmid, phage and IS related genes. Further functional studies are required to understand the full significance of the presence of these genes in these strain genomes. In conclusion, our results showed that both external and internal INDELs causing frame shift mutations and strains specific genes are the major sources of strain divergence in *X. fastidiosa *and possibly for the observed host specificity. On the other hand, functional fingerprinting assays are required to fully understand the significance of the SNPs identified in both coding and non-coding regions.

### Inter-relatedness of the *X. fastidiosa *strains

Analysis of the conserved genes using pair-wise comparison, clearly showed that Temecula-1 has the least deviation (unpaired nucleotide percentage) from the other three strains- 9a5c, Ann1 and Dixon, with 9a5c and Temecula-1 strains being the closest of the three pairs. From Table 2, it can be inferred that the hierarchy of relative similarities between strains is: 9a5c+Temecula-1 > Ann1+Temecula-1 >> Ann1+Dixon > Dixon+Temecula-1 >> 9a5c+Ann1 > 9a5c+Dixon (Table 2). ">>" identifies the degree of observed differences that were supported by ANOVA analysis with Duncan grouping (Table 2). As mentioned previously, the strain comparison was largely influenced by INDELs, with external INDELs being the major type. Similar overall strain relationship conclusion was also observed when internal INDELs alone were compared, with 9a5c showing the least deviation from Ann1 and Temecula-1, 0.039 and 0.044 respectively (Table 2).

However, pair-wise comparisons based on SNPs alone showed a different trend, with the three North American strains showing least strain variability (Table 2). The strain relationship based on SNPs alone can be concluded as: Ann1+ Temecula-1 > Ann1+ Dixon > Dixon+Temecula-1 > 9a5c + Dixon > 9a5c + Ann1 > 9a5c + Temecula-1.

In this study, we systemically compared all the conserved genes in the four strains of *X. fastidiosa*. Our result of inter-relatedness supports the previous whole genome comparison result of three strains of this bacterium [6]. We found that among the 1528 genes that are conserved in all the four strains, the Temecula-1 gene set had the lowest mutation frequency (Table 2) when compared to the other three strain sets, suggesting that Temecula-1 had diversified at a slower rate. Based on this analysis and four-way comparison results showing that Temecula-1 has the lowest number of strain specific genes, it appears that Temecula-1 has undergone the fewest genetic changes of the four strains and may represent the ancestral *Xylella fastidiosa *genome. Interestingly, a close similarity between 9a5c genome and Temecula-1 genome, as revealed by the ANOVA analysis (Table 2) indirectly suggests the possible origination of 9a5c from Temecula-1, as a recent event. Due to the presence of highest number of strain specific genes, we can also conclude that 9a5c is evolving at a faster rate than the other three strains. While the conclusions appear paradoxical, the explanation could be the adaptive, divergence of these strains from an ancestral strain like Temecula, with different rates, as evident by the number of strain specific genes. Further, our results did not show a biogeographic distribution pattern of strain variability, an observation, also made by others [30].

It is interesting to find that the relationship based on total unpaired nucleotide percentage is contrary to that based on SNP. Schuenzel and others [30] constructed a phylogeny tree of *Xylella fastidiosa *using 10 genes. The relationship disclosed by this tree has similarities to our result based on SNP analysis while the result based on total unpaired nucleotide analysis as well as the INDEL analysis differ to their result. This is not surprising as SNPs were the focus of their phylogenetic study and not the INDELs. Further, 9a5c was assumed to be the out-group of the phylogenetic tree; second, sequence information of a limited number of the genes (7) were used for phylogenetic tree construction. As our results indicate, INDELs are the major source of variation in *X. fastidiosa *(Fig. 3), with external INDELs playing a key role (Fig. 4).

### Highly conserved genes among the four strains

Since the 16S or 23S rDNA sequences are well conserved in most organisms, they are frequently used as targets for primer design for PCR detection [31], especially where genome information on the organism is limited. However, currently, there is sequence information on four of the *X. fastidiosa *strains that provides an opportunity to identify other conserved genes. Our analysis shows that while *X. fastidiosa *16S or 23S rDNA sequences are highly conserved among the four strains, there are many other genes that show similar or higher levels of sequence conservation among the four strains and more importantly, are unique to *X. fastidiosa *thereby eliminating the chances of cross contamination or false positives in detection studies (see Additional file 3). This gene list could provide additional reliable primer design targets for *X. fastidiosa *detection and quantification.

### Tandem gene clusters unique to *Xylella fastidiosa*

A similarity search of the coding sequences of the two completely sequenced genomes against the genomes of other sequenced bacteria showed that the 9a5c has 989 genes and Temecula-1 genome has 395 genes that are unique to *X. fastidiosa *with no known homology to any other sequenced bacterial genomes.

Analysis of spatial organization of the above gene sets revealed that in strain 9a5c, 633 genes out of the 989 genes are grouped into 46 gene clusters (~0.523 Mb) (see Additional file 4), while in strain Temecula-1, 282 genes out of 395 are grouped into 23 gene clusters (~0.264 Mb) (see Additional file 5). The average number of genes in each cluster was 13.7 in 9a5c and 12.2 in Temecula-1. The average cluster size was 14.27 kb in 9a5c and 11.49 kb in Temecula-1. As accessed using a BLAST Evalue=e^-05, ^with few exceptions, in majority of the clusters, there was no homology between member genes, which suggest that those clusters are not formed as a result of tandem duplication events (see Additional files 4 &5).

**Figure 6:**
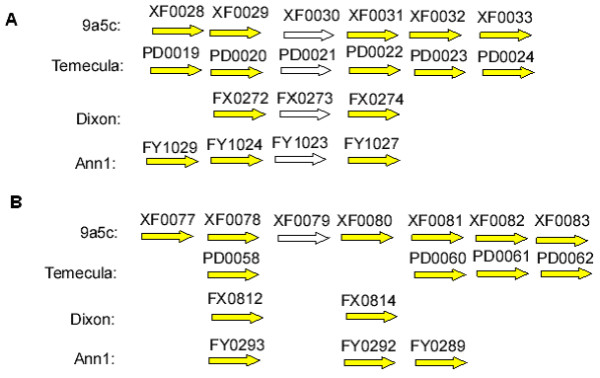
**Organization of the *X. fastidiosa *unique pilin/fimbriae cluster in the four strains**. Yellow color arrows represent genes with clear functions and white color arrows, hypothetical genes. **A Pilin biogenesis cluster**. The ORF XF0028 codes for a pili retraction *PilT *protein, involved in the supply of energy for cell motility [47]; ORF, XF0029 codes for a pre-pili leader sequence; XF0030 is a hypothetical protein; XF0031 codes for the pili assembly protein PilX protein, which is a pilus-associated protein essential for bacterial aggregation and is a key to pilus-facilitated attachment to human cells [48]; XF0032 codes for a *PilY1 *protein, which is thought to be a fimbrial tip-associated adhesion, and involved in fimbrial assembly [49]; XF0033 codes for membrane scaffold protein *PilE *subunits, which are packed to form a hydrophobic core of microtubules [50]. This 7-kb DNA fragment contains genes responsible for type IV pili biogenesis was also found in Temecula-1.**B Fimbriae cluster**. XF0077 and XF0078 are both C chain precursors of type-1 fimbrial proteins while XF0080 is a C chain precursor of type-1 fimbrial protein; XF0079 is a hypothetical protein; XF0081 is an outer membrane usher protein *fimC*; XF0082 is a chaperone protein *ecpD *and XF0083 is the major pilin protein *fimA*.

The majority of the unique gene clusters consists of either hypothetical proteins or phage-related proteins. However, there are a few pathogenicity-related protein gene clusters. A 7-kb region of 9a5c contains six *X. fastidiosa *specific full-length open reading frames (ORFs) involved in pili biogenesis (Fig. 6A). This cluster was first reported in the Temecula-1 strain [32]. Our analysis found that there is a complete match between cluster members of 9a5c and Temecula-1 strains. Each member gene in the 9a5c cluster has a corresponding homologous gene in the Temecula-1 cluster. Further, the relative gene order in these two strains was also conserved. Three genes in this cluster, XF0029, XF0030 and XF0031 were structurally conserved in the draft genomes of Dixon and Ann1 strains, indicating the imperial functions of these genes in pili biogenesis. It is interesting to find that XF0030, a hypothetical protein, is one of the three well-conserved genes. Further functional studies are required to understand its true function. Using the selected criterion described above, our BLASTN results did not identify any homologous sequences for this gene cluster in the GenBank.

Another unique and conserved gene cluster is a 7 kb fimbriae cluster which consists of seven members in strain 9a5c (Fig. 6B). Fimbriae are known to elicit immune responses in eukaryotic cells [33]. Therefore, this cluster might be involved in the invasive activity of *X. fastidiosa *inside host cells. Our analysis shows that XF0078 is highly conserved in the four strains, while XF0080 is missing from Temecula-1 and XF0081 is missing from Dixon strain (Fig. 6B).

Pathogenicity related genes are essential for the movement, colonization and invasion of bacteria inside plant cells. Gene targets that help in the early detection of bacterial colonization and/or invasion would help in designing and implementing better management practices. Our analysis has led to identification of conserved and unique pathogenicity clusters mentioned earlier among the four strains. Targeting the expression of these genes would serve as ideal gene markers for detection and disease management purposes. Previously, Koide *et al *[34], reported XF0078, a fimbrial adhesion precursor, is absent in strain J1a12, a mild strain, compared to the highly pathogenic strain 9a5c, further supporting their potential use as gene markers.

### Putative xenologues *vs *native genes

#### Codon usage analysis

Support trees for hierarchical clustering revealed that native genes and putative xenologues formed distinct groups supported by high bootstrap values. In the case of strain 9a5c, of 36 genes that were identified as putative xenologues based on the BLAST similarity search, 27 genes segregated as a separate cluster consisting exclusively of the xenologues and was supported by a high bootstrap value of 83, from the rest of the genes (see Additional file 6). The other nine putative xenologues formed a subcluster with six native genes. The rest of the native genes formed the third cluster. Among the putative xenologues, the codons, Cys_UGC, Lys_AAG, Gln_CAG, Phe_UUC, Ile_AUC, Asn_AAC, Gly_GGC, Leu_CUG, Arg_CGC and Tyr_UAC were dominantly represented compared to the native genes. Support trees for strain Ann1 showed four clusters, with three clusters for the 25 putative xenologues and the fourth cluster consisting of the native genes (see Additional file 7). While the outer nodes had weak bootstrap values, there was significant support for the inner nodes for all the clusters. Unlike the 9a5c strain, strain Ann1 showed a clear segregation of the native and putative xenologues with various degrees of association in each group. The predominant codons in the putative xenologous group were Ile_AUC, Tyr_UAC, Phe_UUC, Asn_AAC, His_CAC and Cys_UGC. There were seven such putative xenologues in the Dixon strain of which, five formed an independent cluster while the other two (FX0239 and FX3434) paired with the native genes (see Additional file 8). Codons, Lys_AAA, Gln_CAA, Cys_UGC and Phe_UUC were over represented in the transferred genes compared to the native genes. Temecula-1 which has only two putative xenologues was not subjected to this analysis.

#### Base composition GC12 vs GC3 of the putative xenologues

Comparison of the base composition (GC and GC3) between native genes and the putative xenologues showed significant differences at both the positions. Further, for three strains, Ann1, 9a5c and Temecula-1, the GC composition of putative xenologues was higher than in the native genes, while it was lower for the Dixon strain (Table 4).

#### Relative neutrality plots and selection pressures

Relative neutrality plots differed between the native genes and the putative xenologues. While the native genes displayed a slope of 0.649 ± 0.01 among the four strains, the putative xenologues displayed a lower slope in strains Ann1 (0.516), 9a5c (0.295), Dixon (0.487) and on the other hand, strain Temecula-1 showed a higher slope (1.2) (see Additional file 9). The negative intercept observed for strain Temecula-1 might have been biased because it had only few genes.

There are several lines of evidence to show that a sub-set of the strain specific genes identified on the basis of the four-way genome comparison, are truly specific (not present in the other three strains) to that strain and most likely were horizontally transferred into their genome. Such gene transfer events have been documented and are known to influence niche-adaptive variation within *Helicobacter pylori *[35]. However, more complete genome coverage is required to fully understand the potential of such strain specific genes in *X. fastidiosa*. In order to address the issue of draft sequence availability for Ann1 and Dixon strains, in the present study, the strain specific sequences that showed homology to *Xanthomonas *spp. were eliminated thereby reducing the possibility of labeling the genes that are missed in the draft genomes as xenologues. Codon usage analysis has shown that a majority of these xenologues not only show distinct groupings, which could be linked to other bacterial genomic sources, but also show a difference preference for tRNAs species compared to the highly conserved *X. fastidiosa *native gene set. There are several reports in the past which have used codon usage bias to characterize the introduced genes [36,37].

Similarly, significant differences between the native gene set and that of the xenologues were noted for GC composition and the selection pressure existing on the genes compared. Previous analyses of GC composition using relative neutrality plots reasoned that genes undergoing strong selection pressure would tend to show a low slope [36,38,39]. While we did not observe such a trend in our study, the slope differed between native and the xenologous sets in all the four strains, supporting the idea of lateral transfer of these genes.

### Strain specific genomic sequences

Since the aim of the study was also to provide a ready-to-use set of strain specific genomic sequences for developing PCR-based detection techniques for strain differentiation, sequences regardless of function (coding or non-coding), were identified and categorized into four groups (100 -500 bp; 501 – 1000 bp; 1001 -5000 bp and > 5000 bp). There are a total of 1,056, 329, 78 and 293 strain specific genomic sequences for strains 9a5c, Ann1, Dixon and Temecula-1, respectively. Of these, 19 sequences of 9a5c and 16 specific sequences of Ann1 are larger than 1.0 kb.

### Tandem repeats

**Figure 7:**
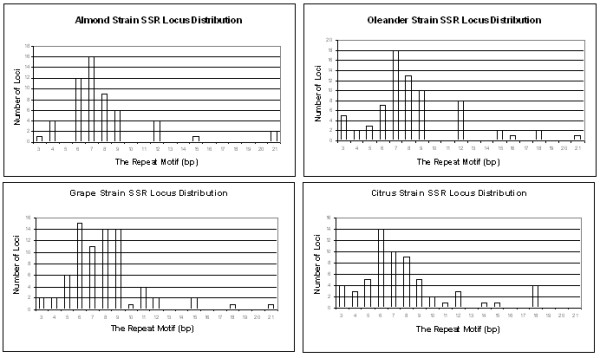
**Distribution of tandem repeat loci in *X. fastidiosa *strains**. The above graphs illustrate perfect and imperfect simple repeats with repeat unit length of 5 bp or more. There were no mono- and di- repeats in all four strains.

There are 84 to 173 tandem repeat sequences with motif size up to 48 bp among the four *X. fastidiosa *strains, with 6 to 9 bp repeat motifs far greater than other motifs (Fig. 7). No mono- or di- repeats were detected in any of the strains in contrast to those present in other bacteria such as *E. coli *[40]. The number of tandem repeat sequences in the non-coding regions varied from 23 to 102 with 1.9 to 40 copies. Similarly, there were 43 to 61 tandem repeats in the coding regions of the four strains, with 1.9 to 74.1 copies. Interestingly, a majority of these motifs came from two categories, hypothetical proteins and proteins associated with phage-related functions, suggesting that further functional characterization will help better understand the epidemiological implications of such repeats in the genomics of *X. fastidiosa *strains.

### Potential problems associated with draft genome sequences

Draft genome sequences provide quick and valuable sequence information at much lesser cost than the complete versions and currently more than 500 microbial draft quality genomes are deposited in the GenBank. While useful information on the sequence content can be derived from the draft genomes, some of the potential problems associated are incomplete sequence information, discontinuity of the sequencing data and greater chances of sequencing and assembling errors. However, in the case of *X. fastidiosa*, we think that problems in the two draft sequences of *X. fastidiosa *are lower due to the fact that more than one strain of *X. fastidiosa *is completely sequenced. Further, *X. fastidiosa *has less repetitive sequences, a problem that compounds in draft genomes with increase in genome complexity (more in eukaryotes in general compared to bacteria) and has no chloroplast and mitochondrial specific sequences. Further, in our analysis, while predicting the potential xenologues in these four strains, we have eliminated genes that are strain specific and show high BLAST similarity to *Xanthomonas *to account for the draft genome status. The published manuscript [6] claimed an error probability of < 1/10,000 for the draft sequences of the Dixon and Ann1 genomes with 9X coverage of the BAC clones, which also meets, by definition the quality of the published CVC and PD complete genomes. While it is impractical to re-sequence the whole genome, we amplified and re-sequenced a small sample set of genomic regions (30 primer pairs targeting INDELs and 10 for frame shift mutations) from the four reference strains 9a5c, Temecula-1, Ann1 and Dixon, that were originally used for genome sequencing. INDELs showed 100% and frame shift mutations showed ~80% agreement with the published genomic sequence in these four strains (data not shown).

## Conclusion

Our four-way genome analysis has identified and characterized conserved and specific genes and non-coding sequences among the four strains of *X. fastidiosa*. Results are presented in a comprehensive database format. The identified variations such as SNPs, INDELs and VNTR are directly applicable for the development of molecular tools for pathogen detection and strain characterization, in understanding disease epidemiology and pathogen biology, and the development of novel disease management strategies.

## Methods

### Custom Perl scripts

Several custom Perl scripts were developed for four-way genome comparisons, such as to search for unique conserved genes (see Additional file 1), highly conserved genes (see Additional file 3), genes with frame shift mutation (see Additional file 2), identify unique gene clusters (see Additional files 4 and 5), and to rank the conserved genes. These program files can be downloaded at our website [41].

### Xfbase structure and contents

Xfbase has been developed under an IIS6.0 server using CGI scripts. Database contents are retrievable through web pages that are dynamically generated by CGI scripts. A total of eight subjects are listed in the index frame: 1. Genome Sequence, 2. Four Way Genome Comparison, 3. Tandem Repeats, 4. Strain Specific Sequences, 5. Unique Genes 6. Conserved Genes, 7. Perl Scripts, and 8. Downloads. Each subject is linked to a corresponding web page which lists the respective data. The data are linked to a web page containing the gene lists. The sequence or alignment information of genes can be conveniently retrieved by clicking the corresponding button next to it. Users can also retrieve the gene information through the provided window in the index frame [41].

### Sequence datasets

Genomic sequences and gene sets of the *X. fastidiosa *strains: *X. fastidiosa *Temecula-1 (grapevine), GenBank #AE009442 and *X. fastidiosa *9a5c (citrus) GenBank # AE003851 were downloaded from the NCBI and the other two strains *X. fastidiosa *Ann1 (oleander) and *X. fastidiosa *Dixon (almond) were downloaded from the Integrative Genomics website [28]. In this database, the gene names of strain 9a5c, Ann1, Dixon, Temecula-1 have prefixes of 'XF', 'FY', 'FX' and 'PD', respectively.

### Four-way whole genome comparisons

The sequence datasets were separated into coding and non-coding regions for analysis. Coding regions for each of the four strains were the same gene sets that were predicted earlier [5-7]. For the non-coding region comparisons, the respective strain gene set was used to mask the complementary region of the whole genome sequence using WUBlast [42] and RepeatMasker [43]. Next, using custom Perl script, all the unmasked regions of at least 100 bp in length were extracted for each strain. Both the coding, as well as non-coding regions were passed through a Perl script pipeline that identified the homologous (conserved) sequences or strain specific sequences based on an arbitrary BLASTN cut-off E-value of e^-10 ^(83% sequence identity).

The homologous pairs of sequences were aligned using ClustalW with default parameters. The regions of variability (SNPs and INDELs) on each line of the alignment were marked with a Perl script (clustalw_modify.pl) to increase the readability. The number of such SNPs and INDELs in each alignment was counted using another Perl script (SNP_Indel_count.pl). From this data, synonymous and non-synonymous SNPs were identified based on the universal genetic code. A custom Perl script (Syndetect.pl) was designed for this purpose. INDELs were estimated according to the Eernisse and Kluge's method [44].

### *Xylella fastidiosa *conserved unique genes and *Xylella fastidiosa *strain specific unique genes

Genes that are conserved in all the four strains but have no known homology to any of the sequenced bacterial genomes were categorized as *Xylella fastidiosa *conserved unique genes. To identify such unique genes, the homologous (conserved) genes identified earlier were used to BLAST search against the GenBank nr database with E-value of e^-5 ^similar to previous report [7].

*Xylella fastidiosa *strain specific unique genes are genes that are present only in a particular strain and have no known homologies to any of the sequenced bacterial genomes. To identify such strain specific unique genes, strain specific genes of individual strains were BLAST searched against the GenBank nr database with an E-value of e^-5^.

### Strain specific genomic sequences

In order to identify the strain specific genomic sequences regardless of their coding status, the genome of each strain was fragmented into 30 bp sequences. These fragments were next added to the Repeat library and each whole genome sequence was masked with the other three strains 30 bp fragments using WUBlast and RepeatMasker programs as described previously. The unmasked genomic sequences were extracted from the result files and grouped into four categories according to their size.

### Pair wise genome analysis of conserved genes

Mapping of the 1,579 conserved genes to their contig position revealed that, 51 genes had truncated start codon due to their end location on the contig and hence were excluded from further analysis. Therefore, only 1,528 conserved genes were used for INDEL, SNP and pair-wise comparisons to determine the inter-relatedness of the four strains. Gene alignments for a total of six such possible groups (9a5c+Temecula-1, Ann1+Temecula-1, Dixon+Temecula-1, Ann1+Dixon, 9a5c+Ann1, 9a5c+Dixon) were generated using CLUSTALW with the default options. For each category, a custom Perl script was used to calculate the number of unmatched nucleotides in the form of SNPs (transition and transversion) and INDELs (external and internal) based on the 1528 sequence alignment files. An unmatched INDEL nucleotide is one that has no corresponding nucleotide in the other gene in the gene alignment file. Mutation frequency was defined as the percentage of unmatched base pairs over the total base pairs aligned and was calculated as reported previously [19]. For each gene, a standard deviation of the mutation frequency among the six compared pairs was calculated and the conserved genes were ranked in descending order of their observed standard deviation.

### Tandem repeats

Sequence repeats were identified for both coding and non-coding regions using Tandem Repeats Finder [45]. The HTML output from this program was converted into plain text and the results were parsed using the tandem_parse.pl Perl script [41]. The complete set of results was tabulated strain wise, with features such as the repeat coordinates, copy number and repeat motif.

### Identification of potential xenologues and defining the native gene set

Homologous searches for each set of the *X. fastidiosa *strain specific genes (9a5c- 241 genes, Ann1- 145 genes, Dixon- 96 genes and Temecula-1 with 10 genes) to the proteins in other sequenced genomes were carried out using BLASTX analysis against the NCBI database using a cut-off E-value of e^-5 ^similar to previous report [7]. The genes showing homology to the plasmid, phage and IS related genes and the *Xanthomonas *genome (a closely related γ-proteobacterium of Xanthomonadales) were removed and the rest of genes from each strain 9a5c (36), Ann1 (25), Dixon (7) and Temecula-1 (2) were treated as potential xenologues and used for further analysis. Out of 1,579 genes that were conserved in all the four strains, the top 100 most conserved genes, based on similarity ranking as described previously, were designated as native genes of that strain.

#### Codon usage and hierarchical clustering analysis

Codon usage analysis for the native genes and the potential xenologues was carried out as described earlier with minor modifications [36]. A matrix file of codon frequencies for each set was generated using the Interactive Codon Usage Analysis (INCA) version 2.0 software [46] and merged into a single file per strain. Roughly, equal numbers of native genes and potential xenologues were used for analysis to avoid statistical bias due to the differences in number of the sequences comprising each set. Support trees for the generated hierarchical clusters were calculated using the TIGR Multiexperiment Viewer (MEV) software with 1000 bootstrap samples.

#### Relative neutrality plots and GC12 *vs* GC3

For each set of the native and potential xenologues, the G+C content at the non-synonymous (GC12) positions was plotted against the G+C content at the synonymous (GC3) position and the slope was determined based on correlation analysis [36].

### Statistical analysis

Statistical significance of the observed mutation rate among the six groups was performed by one-way analysis of variance (ANOVA) using SAS (SAS/STAT software version 9.1, SAS Institute Inc) program and a confidence level of P ≤ 0.05. Duncan's multiple range test was chosen to compare data from different sets.

## Authors' contributions

HD and JY carried out the genome analysis, data interpretation and manuscript preparation. HL conceived of the study and performed the tandem repeat analysis. ELC, MAW and HL participated in the study design, coordination, and data interpretation and helped to draft the manuscript. All the authors read and approved the final manuscript.

## Supplementary Material

Additional File 1 Conserved unique gene sequences. List of *X. fastidiosa *conserved unique genes. Click here for file

Additional file 2 Pair wise analysis of conserved genes with internal INDELs that cause frame shifts. List of conserved genes causing frame shift mutations. Click here for file

Additional file 3 Highly conserved genes. List of highly conserved genes among the four sequenced *X. fastidiosa *strains. Click here for file

Additional file 4 Unique gene clusters in strain 9a5c. List of gene clusters that are unique to strain 9a5c. Click here for file

Additional file 5 Unique gene clusters in strain Temecula-1. List on gene clusters that are unique to strain Temecula-1. Click here for file

Additional file 6 Codon usage analysis. Codon usage analysis of the native and putative xenologues (marked by a colon before the gene name) for 9a5c (Additional File 6), Ann1 (Additional File 7) and Dixon (Additional File 8) strains. Support trees were generated for the hierarchical clusters using the TMEV software with 1000 bootstrap samples. Click here for file

Additional file 7 Codon usage analysis. Codon usage analysis of the native and putative xenologues (marked by a colon before the gene name) for 9a5c (Additional File 6), Ann1 (Additional File 7) and Dixon (Additional File 8) strains. Support trees were generated for the hierarchical clusters using the TMEV software with 1000 bootstrap samples. Click here for file

Additional file 8 Codon usage analysis. Codon usage analysis of the native and putative xenologues (marked by a colon before the gene name) for 9a5c (Additional File 6), Ann1 (Additional File 7) and Dixon (Additional File 8) strains. Support trees were generated for the hierarchical clusters using the TMEV software with 1000 bootstrap samples. Click here for file

Additional file 9 Relative neutrality plots. Relative neutrality plots for native and putative xenologues gene groups in the four *X. fastidiosa *strains. GC12 values are plotted as a function of the GC3 value and the slope of the correlation was determined. Click here for file

## Figures and Tables

**Table 1:** Distribution of strain specific genes among the four strains of *Xylella fastidiosa*.

**Strain**	**Total genes**	**Strain specific**
9a5c	2678	241 (9.0%)
Ann1	2815	145 (5.2%)
Dixon	2622	96 (3.7%)
Temecula-1	2034	10 (0.5%)

**Table 2:** Pair-wise comparison of the 1,528 conserved genes among the four sequenced strains of *Xylella fastidiosa*.

**Strains**	**Bp**	**Paired (%)**	**Unpaired (%)**	**INDEL (%)**	**External (%)**	**Internal (%)**	**SNP (%)**	**Transition (%)**	**Transversion (%)**
9a5c + Ann1	3051309	2951615 (96.733)	99694 (3.267) **a**	56195 (1.842)	55009 (1.803)	1186 (0.039)	43499 (1.426)	6390 (0.209)	37071 (1.215)
9a5c + Dixon	3038700	2915651 (95.951)	123049 (4.049) **a**	82760 (2.724)	80563 (2.651)	2197 (0.072)	40289 (1.326)	5956 (0.196)	34277 (1.128)
9a5c + Temecula1	3047657	2971366 (97.497)	76291 (2.503) **c**	31527 (1.034)	30196 (0.991)	1331 (0.044)	44764 (1.469)	6588 (0.216)	38176 (1.253)
Ann1+ Dixon	3019029	2920562 (96.738)	98467 (3.262) **b**	75229 (2.492)	73471 (2.434)	1758 (0.058)	23238 (0.77)	3224 (0.107)	19939 (0.66)
Ann1+ Temecula1	3047447	2971117 (97.495)	76330 (2.505) **c**	57413 (1.884)	55283 (1.814)	2130 (0.07)	18917 (0.621)	3071 (0.101)	15808 (0.519)
Dixon+Temecula1	3013442	2903990 (96.368)	109452 (3.632) **b**	83880 (2.784)	80452 (2.67)	3428 (0.114)	25572 (0.849)	3812 (0.126)	21704 (0.72)
Average	3036264	2939050 (96.80)	97213 (3.203)	64500 (2.13)	62495 (2.06)	2005 (0.07)	32713 (1.08)	4840 (0.16)	27829 (0.92)

**a, b, c **– Duncan grouping of ANOVA analysis result;

**Table 3:** Pair wise estimation of the synonymous and non-synonymous SNPs among the four sequenced strains of *Xylella fastidiosa*.

**Type**	**Synonymous (percentage)**	**Non-synonymous (percentage)**
9a5c-Temecula1	27174(62.54)	16274(37.46)
9a5c-Ann1	26091(61.88)	16071(38.12)
9a5c-Dixon	23247(60.35)	15272(39.65)
Temecula1-Ann1	10024(56.68)	7660(43.32)
Temecula1-Dixon	14096(57.92)	10243(42.08)
Ann1-Dixon	13097(58.60)	9252(41.40)

**Total**	113729(60.33)	74772(39.67)

**Table 4:** Comparison of the GC composition (total GC *vs *GC at third codon) between native and the potential xenologues gene sets.

**Genes**	**Dixon**		**Ann1**		**9a5c**		**Temecula-1**	
	GC	GC3	GC	GC3	GC	GC3	GC	GC3
Native	0.517 ± 0.03	0.53 ± 0.06	0.512 ± 0.03	0.531 ± 0.58	0.516 ± 0.03	0.5359 ± 0.061	0.514 ± 0.03	0.532 ± 0.059

Xenologues	0.49 ± 0.102	0.49 ± 0.16	0.538 ± 0.05	0.5848 ± 0.09	0.621 ± 0.074	0.76 ± 0.151	0.605 ± 0.063	0.67 ± 0.07

Values given are Mean (± SD) of the GC composition.
