# Supplementary figures and images for: Analysis of the genome-wide variations among multiple strains of the plant pathogenic bacterium Xylella fastidiosa
Source: BMC Genomics. 2006 Sep 1;7:225. doi: 10.1186/1471-2164-7-225 (PMC1574315; doi:10.1186/1471-2164-7-225)

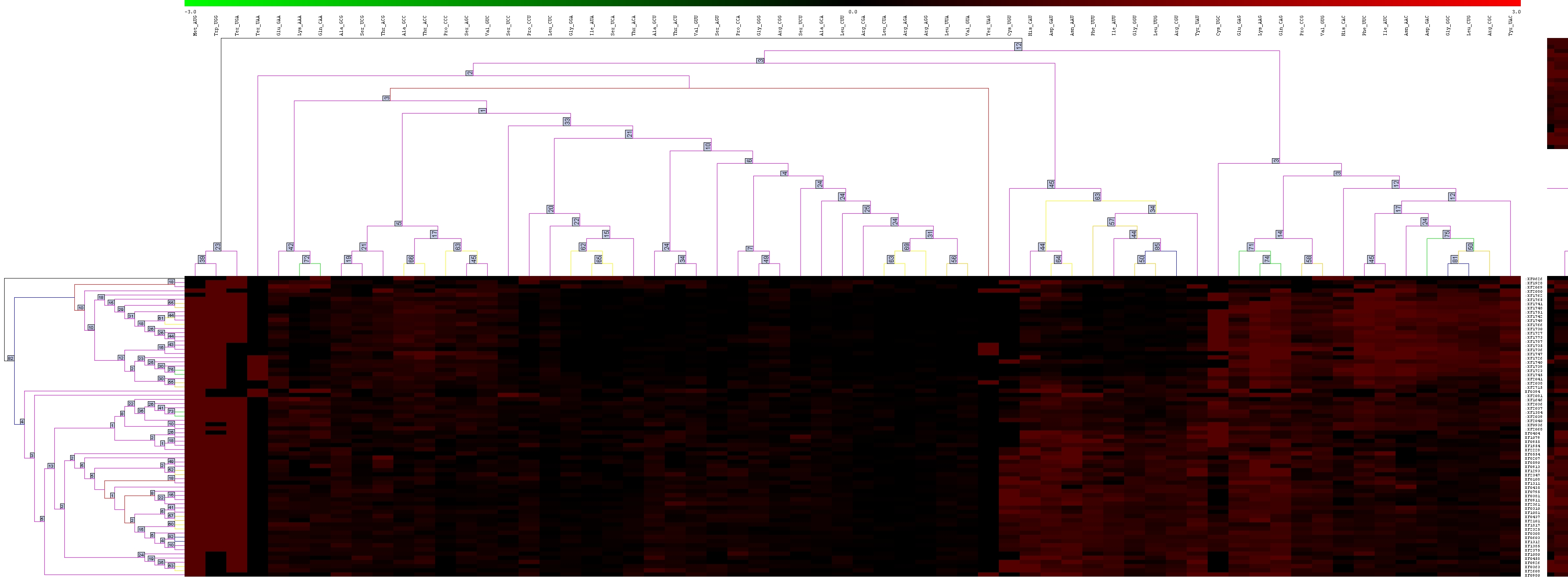

Supplement: Additional file 6 — Codon usage analysis. Codon usage analysis of the native and putative xenologues (marked by a colon before the gene name) for 9a5c (Additional File 6), Ann1 (Additional File 7) and Dixon (Additional File 8) strains. Support trees were generated for the hierarchical clusters using the TMEV software with 1000 bootstrap samples. [file 1471-2164-7-225-S6.jpeg]

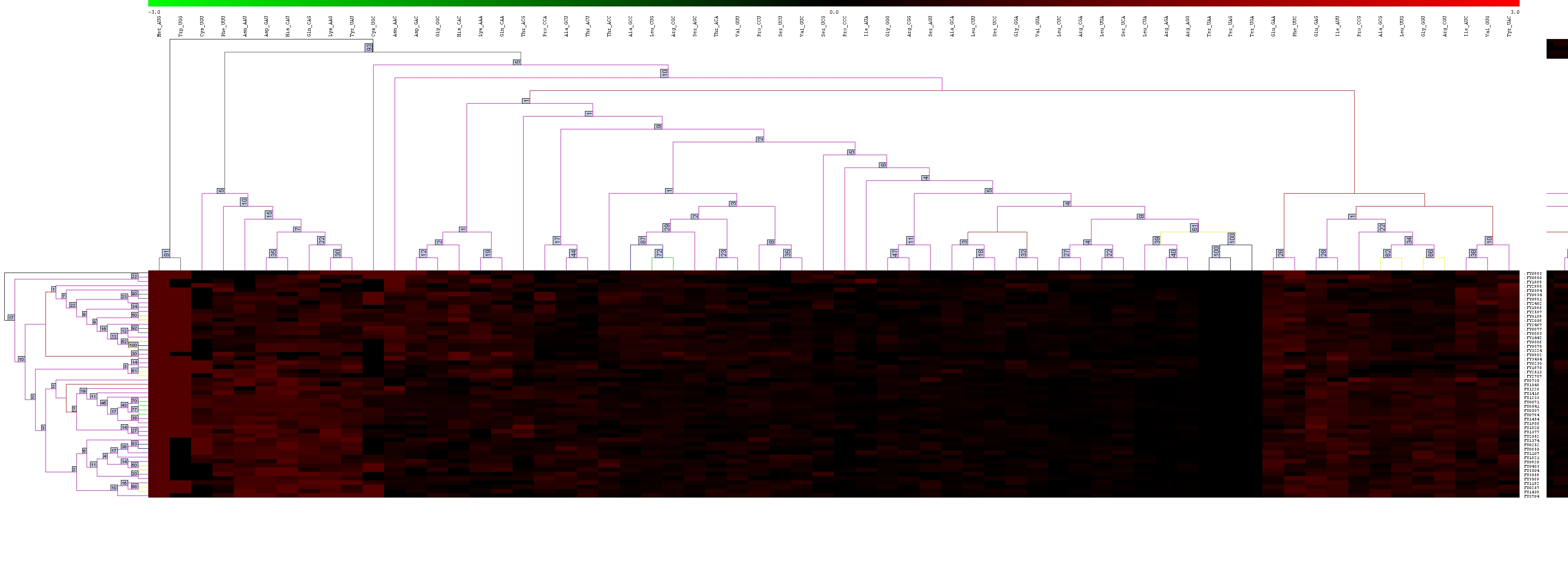

Supplement: Additional file 7 — Codon usage analysis. Codon usage analysis of the native and putative xenologues (marked by a colon before the gene name) for 9a5c (Additional File 6), Ann1 (Additional File 7) and Dixon (Additional File 8) strains. Support trees were generated for the hierarchical clusters using the TMEV software with 1000 bootstrap samples. [file 1471-2164-7-225-S7.JPEG]

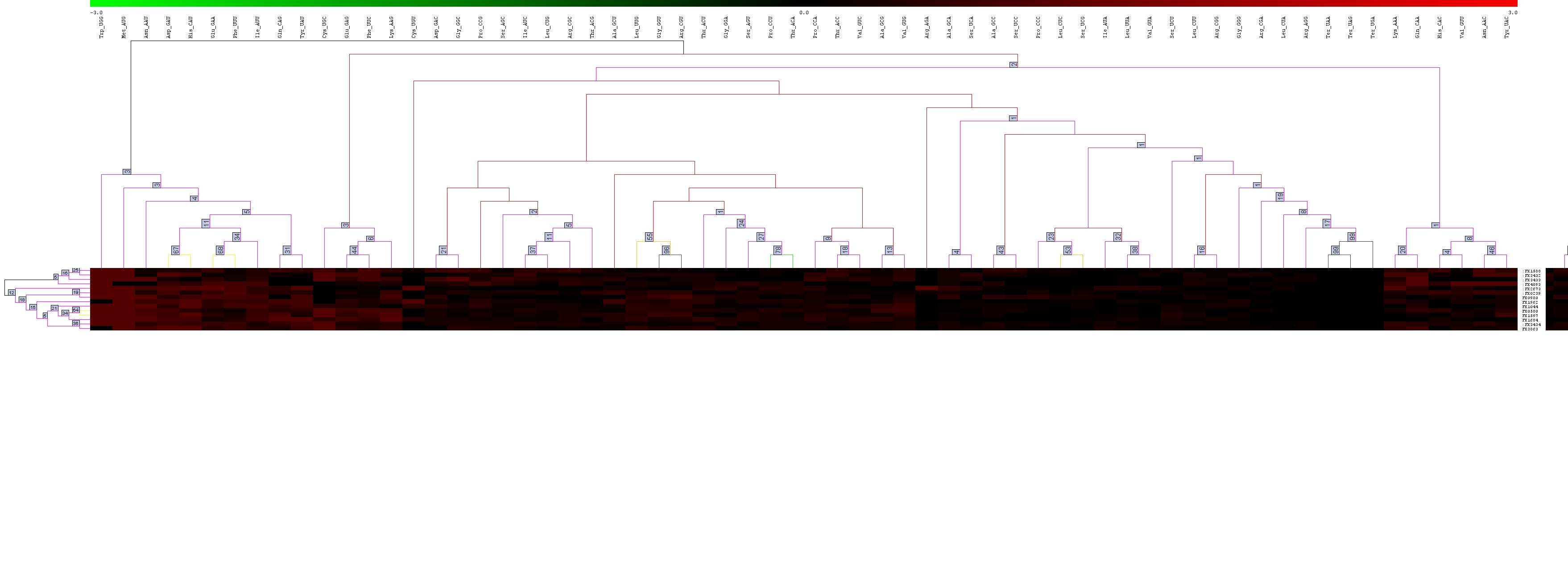

Supplement: Additional file 8 — Codon usage analysis. Codon usage analysis of the native and putative xenologues (marked by a colon before the gene name) for 9a5c (Additional File 6), Ann1 (Additional File 7) and Dixon (Additional File 8) strains. Support trees were generated for the hierarchical clusters using the TMEV software with 1000 bootstrap samples. [file 1471-2164-7-225-S8.jpeg]
