# Supplementary material for: Analysis of the genome-wide variations among multiple strains of the plant pathogenic bacterium Xylella fastidiosa
Source: BMC Genomics. 2006 Sep 1;7:225. doi: 10.1186/1471-2164-7-225 (PMC1574315; doi:10.1186/1471-2164-7-225)
Supplement: Supplementary file 7 [file 1471-2164-7-225-S9.doc]

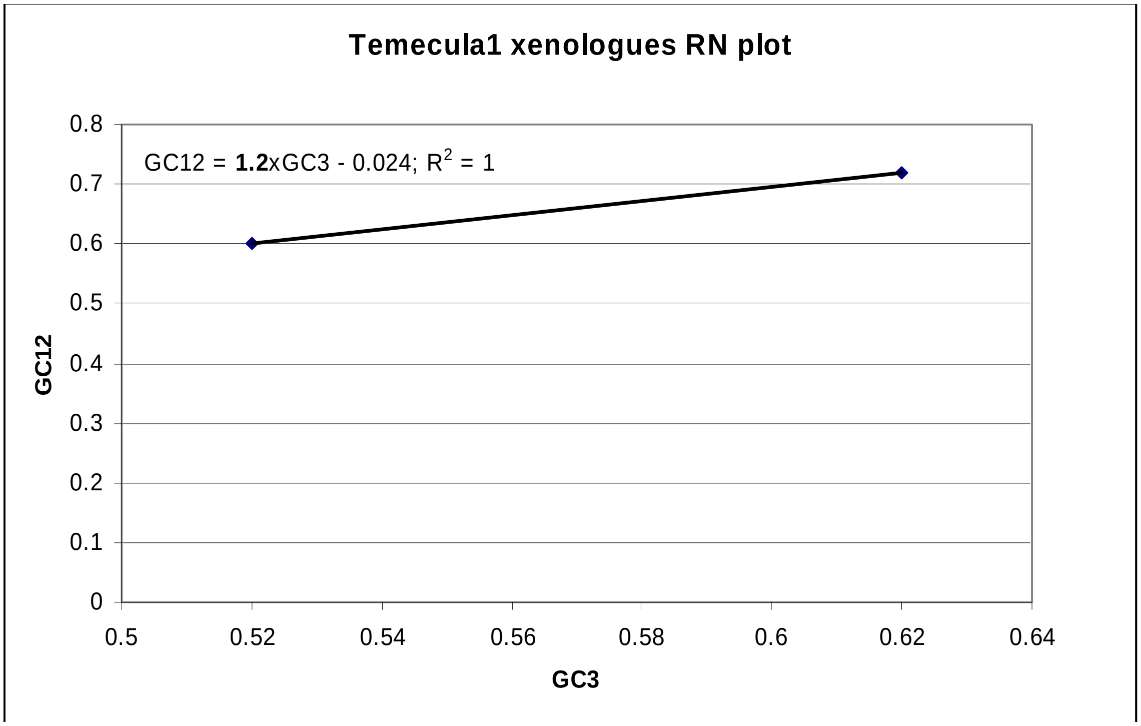
Temecula1 xenologues RN plot
GC12 = **1.2**xGC3 - 0.024; R2 = 1
GC12
GC3
0.8
0.7
0.6
0.5
0.4
0.3
0.2
0.1
0
0.5
0.52
0.54
0.56
0.58
0.6
0.62
0.64

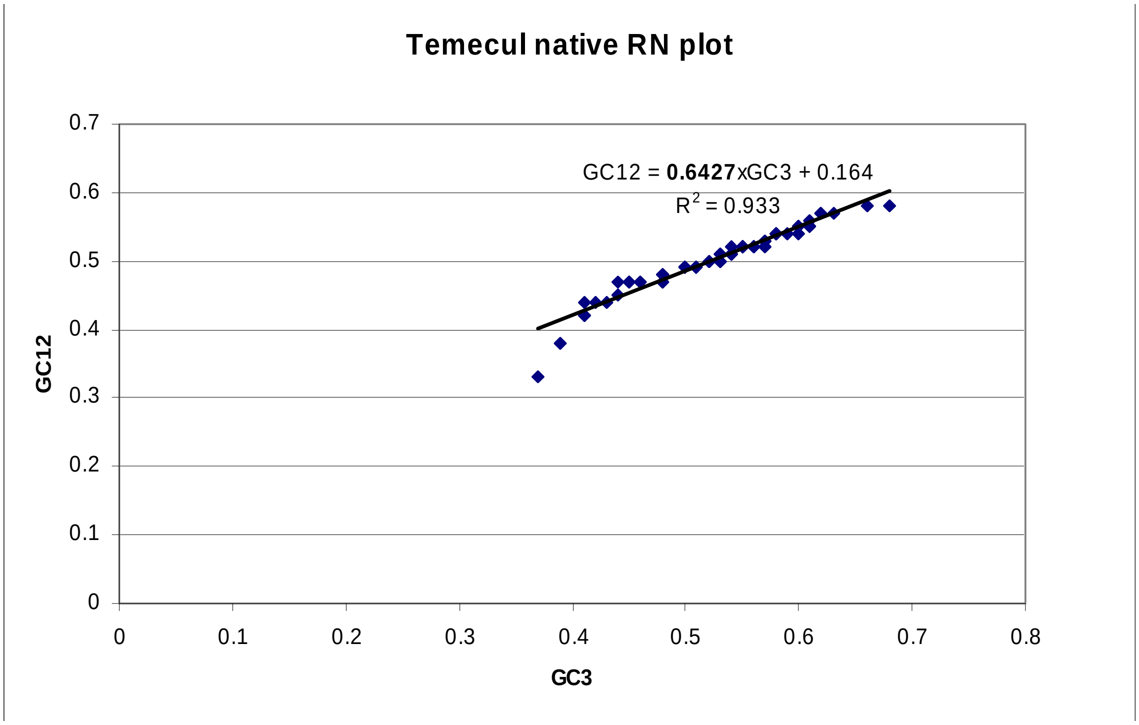
Temecul native RN plot
GC12 = **0.6427**xGC3 + 0.164
R2 = 0.933
GC12
GC3
0.7
0.6
0.5
0.4
0.3
0.2
0.1
0
0
0.1
0.2
0.3
0.4
0.5
0.6
0.7
0.8

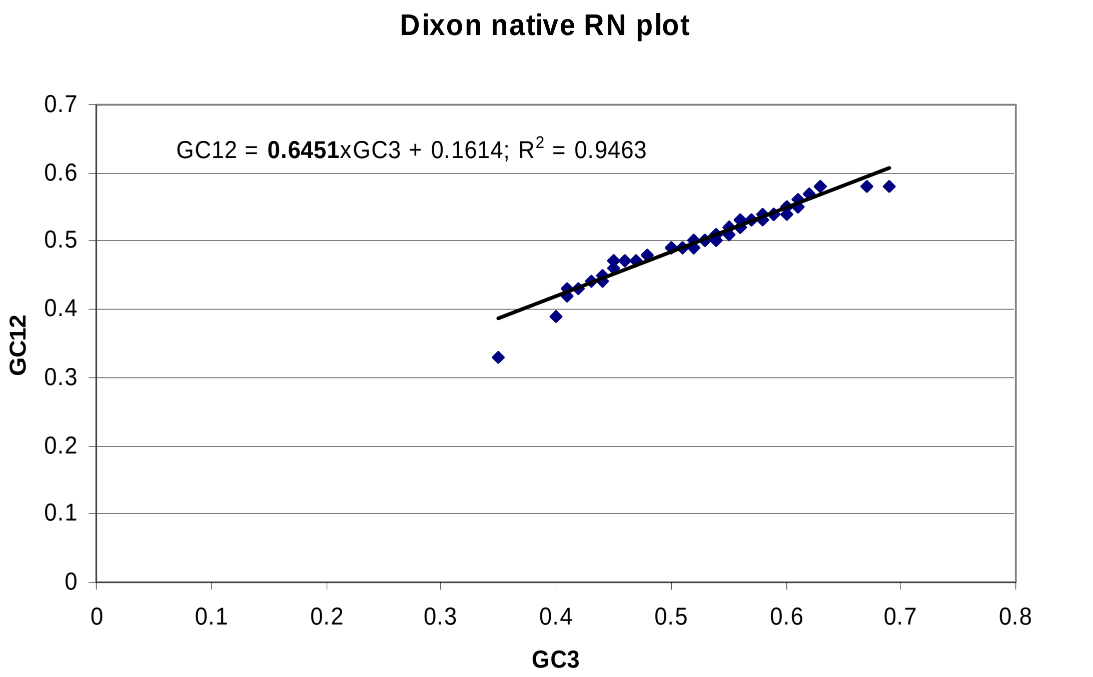
Dixon native RN plot
GC12 = 0.6451xGC3 + 0.1614; R2 = 0.9463
0.7
0.6
0.5
0.4
0.3
0.2
0.1
0
GC12
0
0.1
0.2
0.3
0.4
0.5
0.6
0.7
0.8
GC3

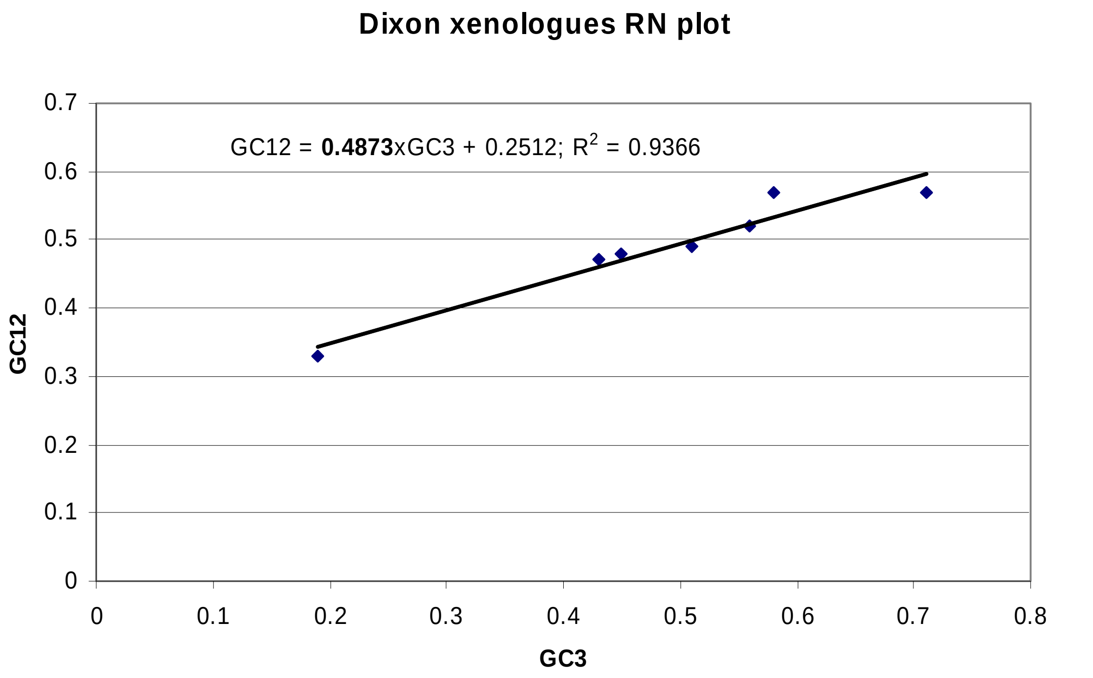
Dixon xenologues RN plot
GC12 = 0.4873xGC3 + 0.2512; R2 = 0.9366
0.7
0.6
0.5
0.4
0.3
0.2
0.1
0
GC12
0
0.1
0.2
0.3
0.4
0.5
0.6
0.7
0.8
GC3

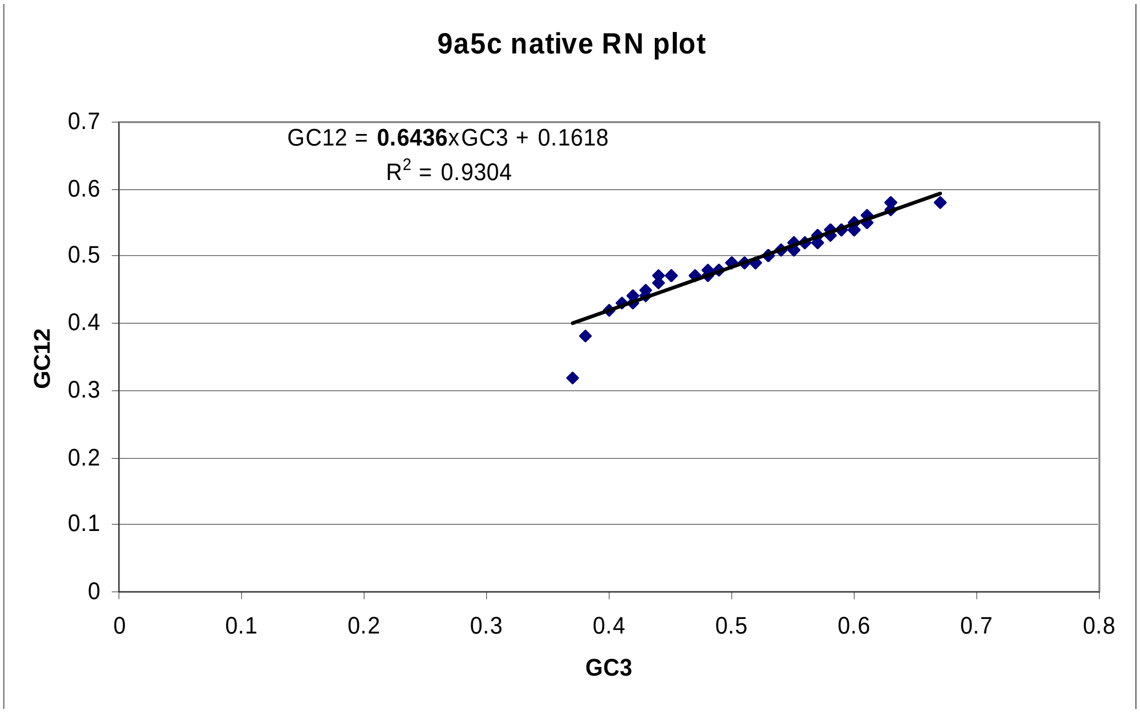

9a5c native RN plot
GC12 = **0.6436**xGC3 + 0.1618
R^2 = 0.9304
GC12
GC3
0
0.1
0.2
0.3
0.4
0.5
0.6
0.7
0.8

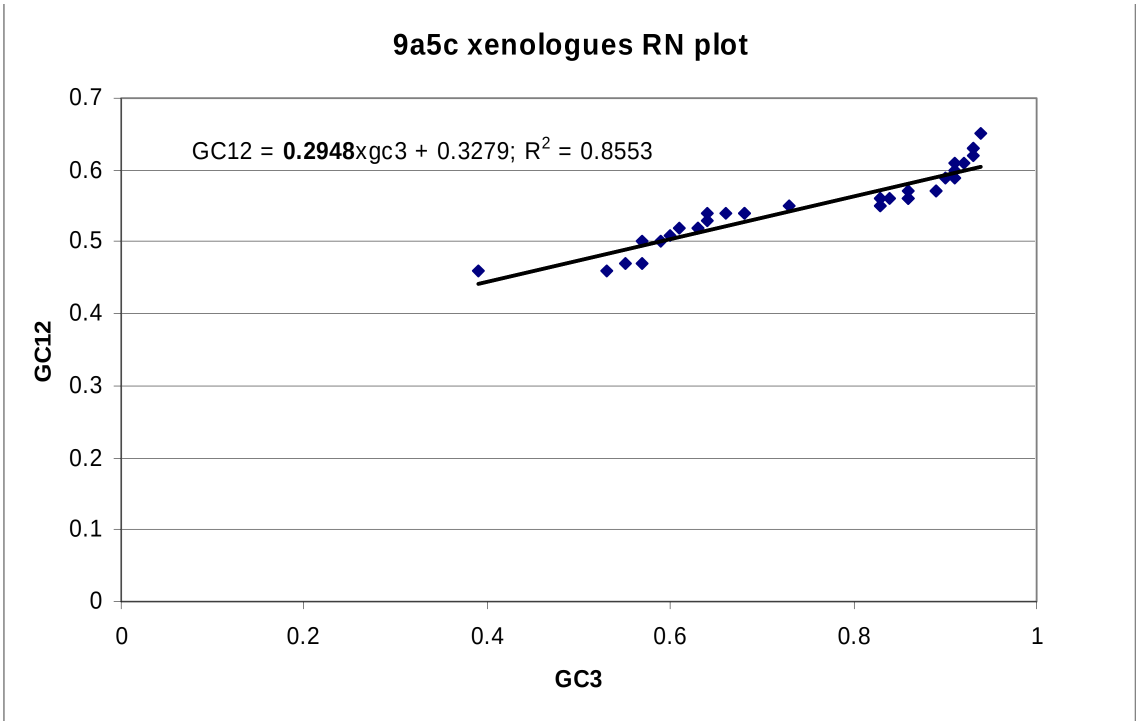

9a5c xenologues RN plot
GC12 = **0.2948**xgc3 + 0.3279; R^2 = 0.8553
GC12
GC3
0
0.2
0.4
0.6
0.8
1

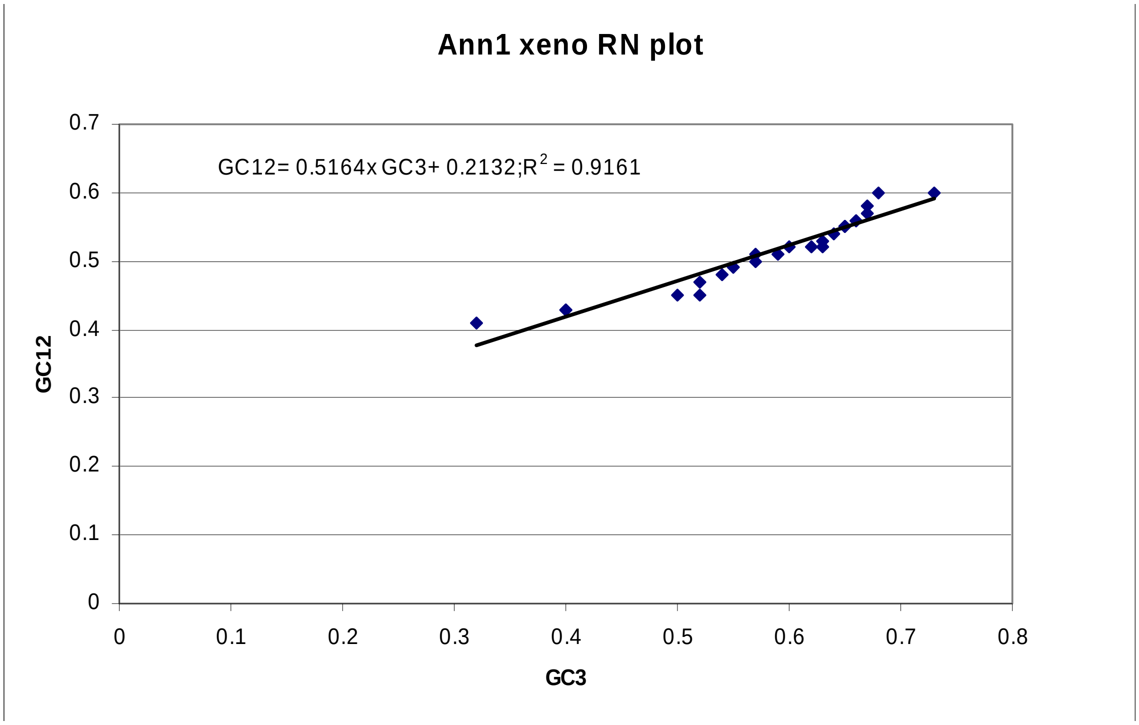
Ann1 xeno RN plot
GC12= 0.5164x GC3+ 0.2132;R2 = 0.9161
0.7
0.6
0.5
0.4
0.3
0.2
0.1
0
GC12
0
0.1
0.2
0.3
0.4
0.5
0.6
0.7
0.8
GC3

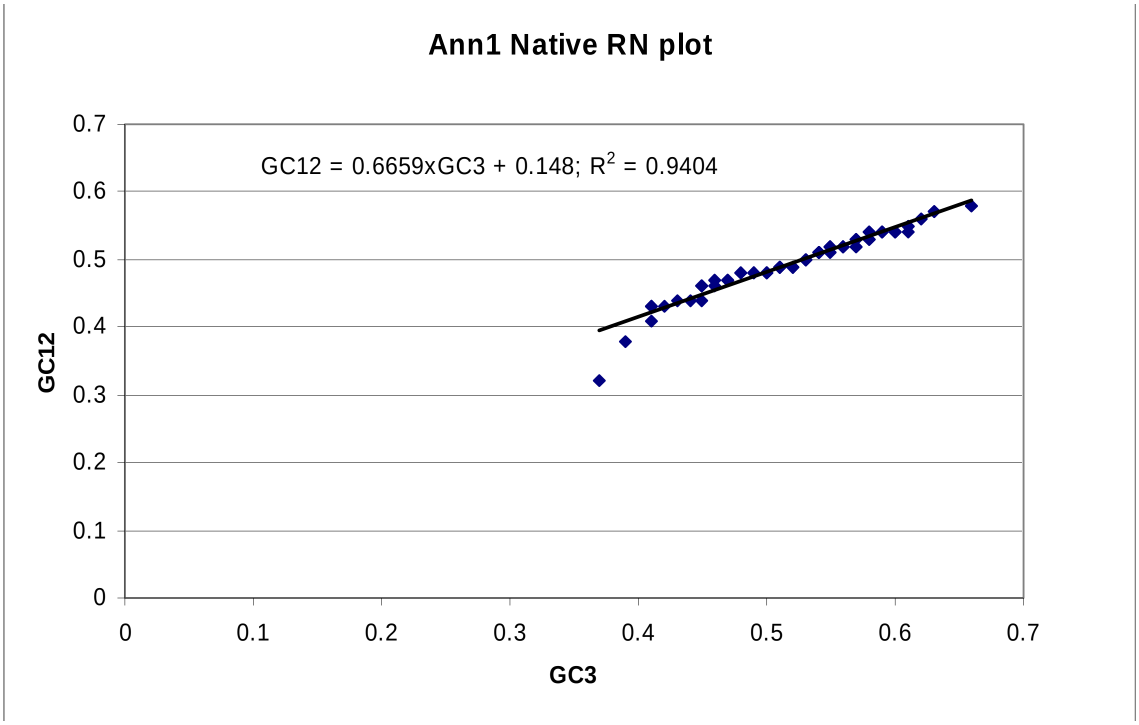
Ann1 Native RN plot
GC12 = 0.6659xGC3 + 0.148; R2 = 0.9404
0.7
0.6
0.5
0.4
0.3
0.2
0.1
0
GC12
0
0.1
0.2
0.3
0.4
0.5
0.6
0.7
GC3
